# Multi-modality approach to detect device-related thrombus after left atrial appendage occlusion: a case report

**DOI:** 10.1093/ehjcr/ytaf363

**Published:** 2025-08-05

**Authors:** Hamady I Maiga, Jana Ambrožič, Borut Jug, Marta Cvijić

**Affiliations:** Department of Cardiology, University Medical Centre Ljubljana, Zaloška 7, Ljubljana 1000, Slovenia; Department of Cardiology, University Medical Centre Ljubljana, Zaloška 7, Ljubljana 1000, Slovenia; Department of Vascular Diseases, University Medical Centre Ljubljana, Zaloška 7, Ljubljana 1000, Slovenia; Faculty of Medicine, University of Ljubljana, Korytkova 2, Ljubljana 1000, Slovenia; Department of Cardiology, University Medical Centre Ljubljana, Zaloška 7, Ljubljana 1000, Slovenia; Faculty of Medicine, University of Ljubljana, Korytkova 2, Ljubljana 1000, Slovenia

**Keywords:** Atrial fibrillation, Left atrial appendage occlusion, Transoesophageal echocardiography, Cardiac computer tomography, Case report

## Abstract

**Background:**

There has been a growing interest in using left atrial appendage occlusion (LAAO) for stroke prevention in patients with atrial fibrillation (AF) who are ineligible for oral anticoagulation. However, device-related thrombus (DRT) may occur after LAAO implantation and poses significant diagnostic and treatment challenges.

**Case summary:**

We describe a case of a patient who suffered an intracranial pontine haemorrhage while on anticoagulation with rivaroxaban for permanent atrial fibrillation and successfully underwent implantation of the LAAO device. Early follow-up transoesophageal echocardiography (TOE) with 3D multi-plane reconstruction revealed a large echo-dense mass on the left atrial aspect of the device. Cardiac computed tomography angiography confirmed a thrombus adherent to the LAAO device. After deciding on the treatment strategy, complete thrombus resolution was achieved at the 6 months follow-up and the patient was free of any thromboembolic and bleeding events.

**Discussion:**

Although DRT is a rare complication following LAAO procedure, an accurate diagnosis is crucial for the specific treatment. Multi-modality imaging approach with TOE and cardiac computed tomographic angiography as complementary methods is helpful to detect complications after LAAO procedure in challenging cases.

Learning points

- Despite growing experience with the left atrial appendage occlusion (LAAO), several serious complications have been reported in LAAO and their management can be challenging.
- While transoesophageal echocardiography is a recommended modality to detect device-related thrombus after LAAO procedure, cardiac computed tomography angiography could be useful as an alternative or additional imaging modality in uncertain cases.

## Introduction

Left atrial appendage occlusion (LAAO) has been proved to be an effective option for stroke prevention in patients with non-valvular atrial fibrillation and contraindications for anticoagulation therapy.^1^ Despite growing interventional experience, serious complications might occur early and late after the procedure. One of these is device-related thrombus (DRT) after LAAO device implantation, which poses significant diagnostic and treatment challenges.^2^

We describe a case of a patient who presented with a DRT early after the insertion of LAAO device.

## Summary figure

**Figure:**
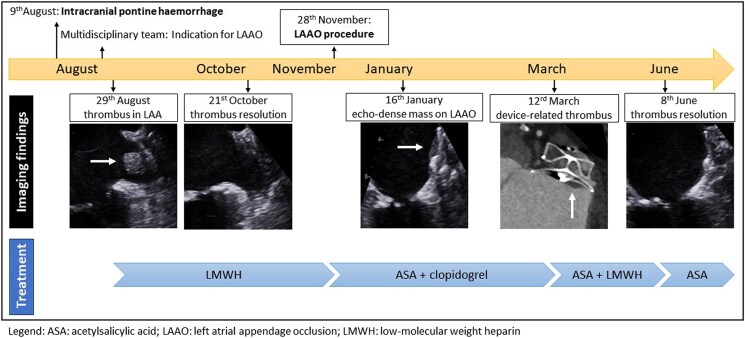


## Case presentation

**Figure 1:**
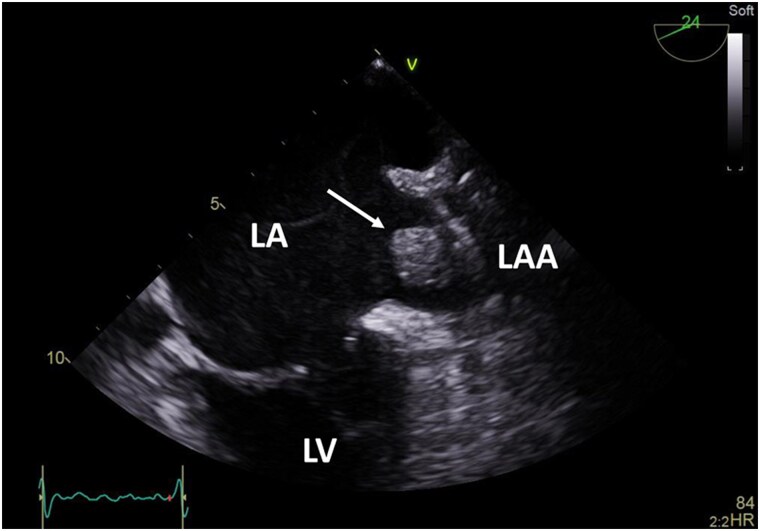
Transoesophageal echocardiography (TOE) of the left atrium. TOE demonstrating large thrombus (2.5 × 1.3 cm) in the left atrial appendage (LAA). LA, left atrium; LV, left ventricle.

A 72-year-old male with a history of prior ischaemic stroke, permanent atrial fibrillation, hyperlipidaemia, diabetes, and recent intracranial pontine haemorrhage while on anticoagulation with rivaroxaban 20 mg daily presented for pre-procedural transoesophageal echocardiography (TOE) before LAAO. His CHA_2_DS_2_-VASc score was 5 and HAS-BLED score of four at that time. On pre-procedural TOE large thrombus in the left atrial appendage was detected (*Figure 1*; Supplementary material online, *Video S1*). After careful consideration and exclusion of ongoing intracranial haemorrhage by brain computer tomography the patient was treated with low-molecular weight heparin (LMWH; dalteparin 7500 units sc twice a day). Three months later total resorption of the thrombus was confirmed, and he underwent transcutaneous implantation of the Amplatzer™ Amulet™ LAA occluder of 31 mm (Abbott, Plymouth, MN, USA). The procedure was uneventful and periprocedural TOE showed intact LAAO device and no evidence of spontaneous echo contrast in the left atrium (see Supplementary material online, *Video S2*). The patient was discharged with his regular antihypertensive therapy and a combination of acetylsalicylic acid 100 mg and clopidogrel 75 mg daily.

**Figure 2:**
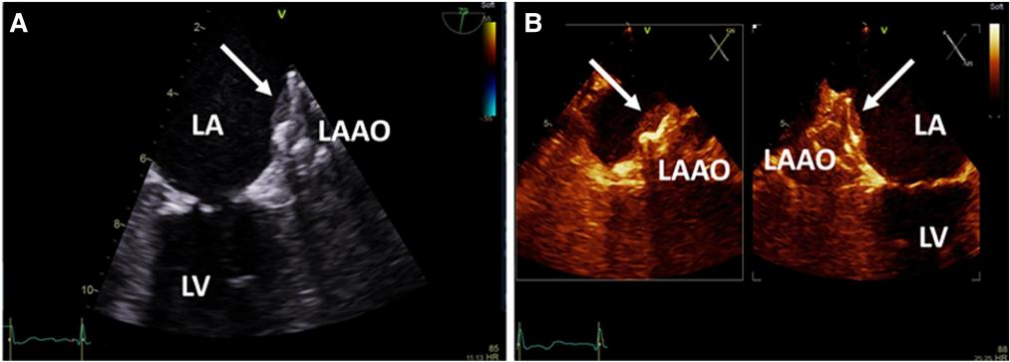
Follow-up transoesophageal echocardiography (TOE) 6 weeks after left atrial appendage occlusion (LAAO) procedure. *(A*) TOE demonstrating an echo-dense mass attached to the LAAO. *(B*) B-mode transforms the image from standard shades of grey to an alternative colour display and was used for better visualization of the mass on the LAAO. LA, left atrium; LV, left ventricle.

At the scheduled TOE 6 weeks after implantation, a good position of the occlusion device with no peri-device residual flow in the left atrial appendage was observed. However, an echo-dense mass on the left atrial aspect of the device, visible in multiple TOE windows, was noted (*Figure 2*; Supplementary material online, *Video S3*).

**Figure 3:**
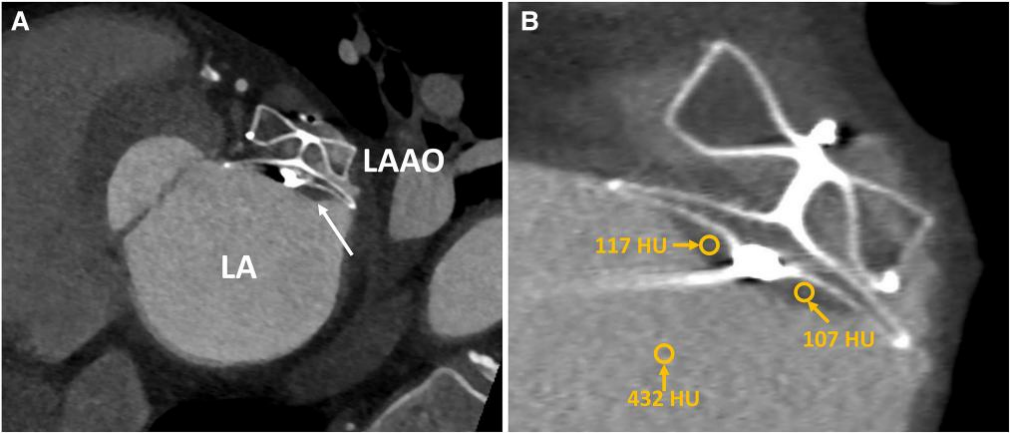
Cardiac computed tomography angiography after left atrial appendage occlusion (LAAO) procedure. *(A*) Hypoattenuated thickening on the atrial device surface consistent with device-related thrombus (DRT) on the device disc. *(B*) Hounsfield unit (HU) measurements of DRT and blood in LA. LA, left atrium; LAAO, left atrial appendage occlusion.

**Figure 4:**
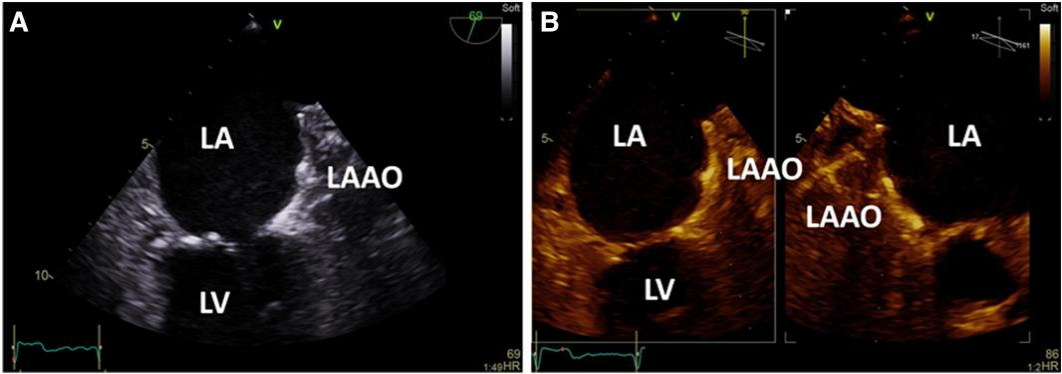
Follow-up transoesophageal echocardiography (TOE) after anticoagulation therapy. TOE demonstrating complete resolution of thrombus on left atrial appendage occlusion (LAAO) on grayscale map (*A*) and B-mode colorization (*B*). LA, left atrium; LV, left ventricle.

3D TOE multi-plane reconstruction revealed a large (1.0 × 1.8 cm, depth 0.4 cm) echo-dense mass at the superior margin of LAAO device (see Supplementary material online, *Video S4*). To better evaluate the mass attached to the occlusion device, we performed a cardiac computed tomography angiography (CTA), which revealed a plane left atrial appendage filling defect with a surface area of 2 cm^2^ and a width of 0.4 cm in contact with the device that was consistent with a thrombus (*Figure 3*). The patient was restarted with LMWH (dalteparin 7500 units sc twice a day) while continuing with acetylsalicylic acid daily for 3 months. At follow-up complete thrombus resolution was achieved (*Figure 4*; Supplementary material online, *Videos S5* and *S6*). The patient was maintained on monotherapy with acetylsalicylic acid 100 mg daily and he had no additional ischaemic or bleeding events.

## Discussion

LAAO is generally accepted as a therapy for patients at high risk of AF-related thromboembolism who are not able to tolerate long-term treatment with oral anticoagulation.^1^ Thrombus on the occlusion device is uncommon, but serious complication, usually occurring early after LAAO procedure.^2^ DRT was described in 3.7% to 7.2% in recent large series^2^ and is associated with a higher rate of stroke and systemic embolism.^3^ The exposed device surface and incomplete endothelialization are prone to thrombi formation. As complete endothelialization is expected several weeks after device implantation, the incidence of DRT is highest in the early post-implant period.^4^

Several factors associated with increased risk of device thrombosis have been recognized, such as hypercoagulability disorder, iatrogenic pericardial effusion, deep device implantation, non-paroxysmal atrial fibrillation and a history of thromboembolism.^3^ The last two risk factors were also present in our patient. Additionally, our patient had left atrial appendage thrombosis previously. There is some conflicting data on the association between the type of the device or short-term anticoagulation/antithrombotic therapy after LAAO procedure and the occurrence of DRT. However, latest meta-analysis did not show differences in DRT rates between the Amplatzer and Watchman devices.^4^

Although TOE has been traditionally the gold-standard post-procedural imaging modality for LAAO device,^5,6^ artefacts from the device or surrounding structures may limit visualization of the LAAO surface and consequently detecting DRT. In some cases, an imaging artefact could be mitigated by probe manipulation and comparison with the post-implantation TOE. The following five echocardiographic diagnostic criteria were proposed for the diagnosis of DRT on TOE: an echo density on the left atrial aspect of the device not explained by imaging artefacts; inconsistency with normal healing or device incorporation; visible in multiple TOE planes; in contact with the device; and exhibiting independent motion.^7^ Contrast echocardiography can also be used to facilitate thrombus detection by providing contrast opacification within the left atrium to clearly show the filling defect of a thrombus on the device disc. In challenging cases of detecting LAAO complications, CTA may be the preferred imaging modality due to its superior spatial resolution and detailed tissue characterization.^2,8^ The identification of DRT on CTA is based on the observation of hypoattenuated thickening on the atrial device surface. Although the Hounsfield attenuation value may be recorded, its clinical value remains undetermined.^9^ On the contrary, cardiac magnetic resonance is not the optimal imaging modality due to device-related imaging artefacts.

Currently, there is no consensus on the optimal treatment regimen of DRT and the duration of therapy. In most studies, the patients were treated with LMWH, intravenous heparin or oral anticoagulation.^10^ Reported duration of antithrombotic treatment was from 2 weeks to 6 months. Complete thrombus resolution with initiation of anticoagulation therapy was achieved in 74.7% to 97% of patients^4,10,11^ with recurrence rate of 35% during a median follow-up period of 15 months after cessation of anticoagulation therapy.^12^ Our patient was treated with LMWH for 3 months when resolution of thrombus was demonstrated while continuing to receive acetylsalicylic acid. Follow-up is needed to exclude thrombus recurrence while receiving only antiplatelet therapy.

## Conclusion

After LAAO procedure careful TOE follow-up is mandatory for detecting LAAO related complications and when in doubt multi-modality imaging should be used. CTA has important added value in the detailed characterization of the DRT and confirmation of the diagnosis. A short course of antithrombotic therapy is usually prescribed after diagnosing DRT, if tolerated by the patient.

## Supplementary Material

ytaf363_Supplementary_Data

## Data Availability

The data underlying this article are available in the article and in its online Supplementary material.

## Lead author biography

After graduating from the Faculty of Medicine at the University of Ljubljana, Slovenia, Dr. Hamady I. Maiga began work as a cardiology fellow at the University Medical Centre Ljubljana. His area of interest is echocardiography and multi-modality imaging.
